# Identifying Elements for a Cardiac Rehabilitation Program for Caregivers: An International Delphi Consensus

**DOI:** 10.3390/healthcare12202049

**Published:** 2024-10-16

**Authors:** Maria Loureiro, João Duarte, Eugénia Mendes, Isabel Oliveira, Gonçalo Coutinho, Maria Manuela Martins, André Novo

**Affiliations:** 1 School of Medicine and Biomedical Sciences Abel Salazar, Cintesis-NursID, 3000-602 Coimbra, Portugal; 3028@chuc.min-saude.pt; 2 Coimbra Local Health Unit, 3000-602 Coimbra, Portugal; 7220@ulscoimbra.min-saude.pt (J.D.); 8058@ulscoimbra.min-saude.pt (G.C.); 3 LiveWell-Research Centre for Active Living and Wellbeing, School of Health, Polytechnic Institute of Bragança, 5300-121 Bragança, Portugal; maria.mendes@ipb.pt; 4 Nursing School of Coimbra (ESEnfC), The Health Sciences Research Unit: Nursing (UICISA:E), 3004-011 Coimbra, Portugal; isabel.oliveira@esenfc.pt; 5 Faculty of Medicine, University of Coimbra, 3000-602 Coimbra, Portugal; 6 Nursing School of Porto, Cintesis-NursID, 4200-450 Porto, Portugal; mmartins@esenf.pt

**Keywords:** caregivers, cardiac rehabilitation, heart disease, prevention, Delphi

## Abstract

**Background/Objectives:** Caregivers of patients with heart disease may often feel physically, emotionally, and psychologically overwhelmed by their role. The analysis of cardiac rehabilitation (CR) components and caregivers’ needs suggests that some interventions may benefit them. Therefore, this study aimed to identify a consensus on the CR components targeting caregivers of patients with heart disease. **Methods**: A three-round international e-Delphi study with experts on CR was conducted. In round 1, experts provided an electronic level of agreement on a set of initial recommendations originating from a previous scoping review. In round 2, experts were asked to re-rate the same items after feedback and summary data were provided from round 1. In round 3, the same experts were asked to re-rate items that did not reach a consensus from round 2. **Results**: A total of 57 experts were contacted via e-mail to participate in the Delphi panel, and 43 participated. The final version presents seven recommendations for caregivers of patients with heart disease in CR programs. **Conclusions:** These recommendations are an overview of the evidence and represent a tool for professionals to adapt to their context in the different stages of CR, integrating the caregiver as a care focus and as support for their sick family members. By identifying the components/interventions, there is potential to benchmark the development of a cardiac rehabilitation strategy to be used and tested by the healthcare team for optimizing the health and role of these caregivers.

## 1. Introduction

Heart disease is one of the most significant challenges in the world today and one of the leading causes of death worldwide, with high economic and health impacts [1]. Cardiac rehabilitation (CR) is considered a fundamental part of the intervention in these diseases.

CR programs are multidisciplinary, multifactorial secondary prevention interventions usually consisting of structured physical exercise, psychological support, and education. They promote positive lifestyle changes and control risk factors in patients with heart disease [2,3]. They are traditionally divided into three phases: phase 1 (inpatient), phase 2 (outpatient), and phase 3 (in the community). Intervention methods can be face-to-face or, more recently, resort to telehealth [4,5]. The core CR components/interventions are patient assessment, exercise training, dietary counseling, risk factor management (smoking, lipids, blood pressure, weight, and diabetes mellitus), and psychosocial intervention [6]. The advantages of CR include reduced lengths of stay and re-hospitalizations, reduced healthcare costs, and increased exercise capacity, quality of life, and psychological well-being [7,8]. Despite its benefits, CR remains underused, with less than 50% of eligible patients participating in such programs due to barriers related to patients, healthcare professionals, and healthcare systems [9]. Those related to the patients are mainly the need for literacy, the family/social environment, and adherence to the therapeutic regime of medication, diet, and exercise.

Caregivers contribute to these patients’ recovery in many ways. They facilitate activities of daily living; provide emotional support; ensure adherence to and management of the therapeutic regimen [9,10]; provide symptom control, health monitoring, and encouragement; and participate in physical activity and exercise. Caregivers can be agents for improving adherence to CR programs. Some studies have shown that positive caregiver support can help facilitate recovery and adjustment following an acute cardiac event [10].

Caregivers of patients with heart disease represent a subgroup of caregivers who often feel physically, emotionally, and psychologically overwhelmed by their role. They can experience sleep disturbances, high levels of fatigue, as well as an increased risk of developing heart disease and death [11,12,13]. The burden of caring for their sick family members can lead to the neglect of their health and risky behaviors, such as poor eating habits and a sedentary lifestyle. These caregivers should, therefore, be the target of intervention by health professionals, not only in training for the role of caregiver but also in monitoring their health [14].

Although some CR programs often include caregivers in educational sessions, they receive less information than the patients, and the focus is usually on improving patient outcomes [10].

By examining the components of CR and assessing the needs of caregivers, it appears that integrating these caregivers into rehabilitation programs could bring significant benefits, generating a symbiosis of simultaneous gains for the dyadic of patients with heart disease and caregivers. It, therefore, seems essential to develop a consensus on CR interventions/components that can improve health and enhance the roles of caregivers of patients with heart disease.

The research question was “Which cardiac rehabilitation interventions/components can be implemented for caregivers of heart disease patients?”. The results from a previously published scoping review supported this study [15]. This scoping review aimed to map the interventions directed to the caregivers of patients with heart disease in CR programs.

Three separate domains were analyzed: the population, the context, and the concept. The population was caregivers of patients with heart disease, the concept was components/interventions of CR programs (e.g., educational intervention and exercise prescription), and the context was all settings (hospital, home, and community).

The results of previous scoping reviews suggested that including specific interventions for caregivers improved their health and empowered them, increasing the quality of care provided to both the caregiver and the patient.

This study contributed to identifying important issues to facilitate the development of evidence-based cardiac rehabilitation, identify gaps, build knowledge, and inform other studies [15].

## 2. Materials and Methods

A modified Delphi study (RAND/UCLA Appropriateness Method) was developed [16] for its efficiency and ability to invite geographically distant experts. The Delphi method provides the opinion of a group of experts, called a panel, on a subject in a structured format in which interaction between different members occurs through a questionnaire. Due to the use of the Internet, several authors suggested calling this e-Delphi or modified Delphi, as online platforms replaced physical questionnaires. The advantage of this method is that it allows more people to participate by eliminating geographical distance and maintaining the anonymity of participants, avoiding any influence on the responses of panel members [16,17,18]. Furthermore, it is relatively cheap and helps organize and import data for database analysis.

This study took place between March 2023 and August 2023. Consistent communication with reminder e-mails was sent out automatically every week for up to 3 weeks, and one reminder e-mail was sent three days before each survey expired.

Cardiologists, nurses, rehabilitation nurses, physiotherapists, nutritionists, exercise physiologists, and clinical psychologists were invited to participate to enhance depth and diversity. The invitation to these experts follows the recommendations that identify the professionals who should be part of the CR program teams [19]. In addition to these professionals, researchers with published evidence in CR were also invited to participate. The sampling technique was non-random and convenient. After identifying the experts from publicly available professional and academic profiles, they were invited by email to join the panel [20].

To determine the number of experts to invite, evidence shows that it can vary from 3 to 713, with an average of 14 [21]. Attrition is expected through the sequential rounds. Evidence shows that not all experts in Delphi panels participate in all rounds, varying between 35% and 87%, so it is advisable to invite a minimum of 30 experts to guarantee expressiveness in the responses [16,17]. This value was used as a minimum reference, and close to twice as many were invited, with the expectation that some might not respond; in total, 57 experts were invited.

The Delphi is at greater risk for attrition when compared to other research methods because it typically involves two to three rounds, which can cause expert fatigue, distraction between rounds, or discouragement with the Delphi research method [21]. The expectations and instructions for the e-Delphi study were meticulously communicated during the informed consent process before the first round, aiming to minimize attrition and foster strong commitment among the experts.

Each panel member signed an online consent form and completed the subsequent surveys via a secure link sent to their professional e-mail addresses. Confidentiality of data by the research team and anonymity between experts were maintained throughout the study using the protected online survey platform (Microsoft Forms—https://forms.office.com/). Only the principal investigator had access to password-protected survey research. 

A maximum of three rounds was defined in which participants were asked to comment on the characteristics of the caregivers to integrate into CR programs, the components/interventions of the programs, the contexts in which these programs should be developed, and a final statement on the level of agreement of CR as a primary intervention for the caregivers. The first round consisted of a set of statements developed by the research team, guided by the literature identified in a previous scoping review [15]. The experts were asked to analyze the components/interventions that can be included in the care of caregivers of patients with heart disease.

The level of agreement for each statement in the first two rounds was measured using a Likert scale ranging from 1 to 5, with the following values assigned to each number: 1—Strongly Disagree; 2—Disagree; 3—Neither agree nor disagree; 4—I agree; 5—Totally Agree. Based on the evidence, the participants were asked to justify lower degrees of agreement and to add commentaries if needed. In the third round, each statement’s degrees of recommendation were agreed upon. There is no clear consensus on the criteria for acceptance or rejection in Delphi studies [22,23]. Despite this, the researchers established the criterion that only statements with a degree of agreement higher than 75% would be included, i.e., statements with the sum of agreement of “4” or “5” on the Likert scale would go on to the next round. The degree of recommendation followed the proposal of the ACC/AHA Clinical Practice Guideline Recommendation Classification System [24]; Class I were all the statements with ≥75% of “Totally agree”, Likert-5, and Class II were all the others responses. A maximum time limit of four weeks was set for responding to each round.

Round 3 assessed the extent of agreement on the level assigned to each formulated recommendation.

Descriptive statistical analysis was used to provide an understanding of the degree of consensus. The qualitative feedback from the comments of the first round was categorized, used to formulate new statements, and then shared with the participants to solicit additional judgments and/or achieve consensus where necessary.

## 3. Results

Of the 57 invited, 43 experts agreed to participate (75.44% response rate), with a mean time of 15 ± 9.72 years of experience in CR practice/research. Twenty countries were represented, with experts from 10 professions (Table 1).

Despite the different representations in both countries and professionals, there were no significant differences in responses throughout the rounds.

In the first round, based on nine initial statements developed by the research team, three new recommendations were added, two were proposed for revision, and three were rejected. The proposed changes from the first round were agreed upon during the second round. In the third round, the recommendation levels for each statement were established (Table 2). Finally, in the fourth round, the degree of agreement on the recommendation categories, defined according to the evidence and outcomes from round two, was confirmed. This process resulted in seven recommendations for interventions, components of CR, and location/phase considerations for caregivers (Table 3).

### 3.1. Domain 1—Population

Domain 1 explored the population of caregivers of patients with heart disease. The experts’ feedback was that the patient/caregiver dyad should be included in CR programs, regardless of the cardiac disease of the person treated, if CR is indicated. In domain 1, experts strongly agreed (88.85%). They considered a Class II recommendation: “Caregivers of patients with heart disease may benefit from cardiac rehabilitation programs and, therefore, should be encouraged to participate alongside the heart disease patient they support in cardiac rehabilitation programs”.

Experts also strongly agree (100%) that integrating caregivers can increase the level of adherence to CR programs, and it was considered a Class I recommendation: “The integration of caregivers in cardiac rehabilitation programs is likely to increase the adherence of cardiac patients to programs and, therefore, should be encouraged”.

### 3.2. Domain 2—Concept

Domain 2 explored the components/interventions of CR programs (e.g., educational intervention and physical exercise prescription).

The “physical exercise prescription” component was excluded in the first round, with an agreement rate of 65.2%. Experts considered it essential but challenging to implement, considering the need for individualization and the constraints of patients to access this CR component.

Regarding “Educational intervention”, experts strongly agreed (97.45%), which was considered a Class I recommendation. “Caregivers of patients with heart disease may benefit from health education sessions comprising information about the disease, nutrition, physical activity, and basic life support training”. Training in basic life support for caregivers is a specific area of the educational intervention.

About the intervention “Risk factor management”, experts strongly agreed (82.8%). It was considered a Class II recommendation: “Caregivers of patients with heart disease and patients likely share cardiovascular risk factors and, therefore, they may also benefit from participating in the patients’ risk factor management”.

Although CR is considered a post-event secondary intervention for caregivers, it can be understood from a primary prevention perspective, aiming to improve the health status of caregivers. The panel of participants strongly agreed (83.9%) that “Cardiac rehabilitation can be helpful as a primary prevention intervention for heart disease patients’ caregivers”, and it was considered a Class II recommendation.

### 3.3. Domain 3—Context

Domain 3 explored the context of the intervention (hospital, home, community care), or care delivery methods (presential, online). Most published studies that included caregivers were home-based programs. However, experts in the first round (agreement of 58.7%) did not agree to recommend home-based programs as the most suitable for integrating caregivers.

Regarding the CR phases, phase 1 (inpatient) was not included in the recommendations as there is no evidence supporting the integration of the caregivers in this phase. The panel accepted the inclusion of caregivers in phases II and III with strong agreement (93%). It was considered a Class II recommendation: “Phases II and III of cardiac rehabilitation may be the most suitable moments to integrate caregivers into these programs”.

In-person intervention methods are the most widely studied. However, when the adherence data was analyzed, the recommendations evolved towards telerehabilitation. Therefore, the panel agreed that this method could facilitate the use of CR by caregivers with strong agreement (82.3%). It was considered a Class II recommendation: “Telerehabilitation may be a method to facilitate the integration of caregivers in cardiac rehabilitation programs”.

The final version presents seven recommendations for caregivers of patients with heart disease in CR programs (Table 3).

## 4. Discussion

This expert consensus addresses an area of scarce evidence, intending to integrate and improve care, and is a guideline for clinical practice and research in this field. This research integrates all caregivers of patients with heart disease as important care targets, especially in the outpatient/community area. It also highlights the educational and risk factor control components of CR programs as the most suitable for caregivers. Telerehabilitation appears to be a methodology that can be used with caregivers. This panel reinforces the positive impact of integrating caregivers into CR programs on the adherence of patients with heart disease and the health of the dyadic.

Forty-three experts from 20 countries comprised this panel, a higher number than a previous Delphi held on CR programs by Ambrosetti and colleagues in 2021 [25]. A more significant participation of international experts in studies of this nature suggests a growing interest in CR.

Integrating caregivers into CR programs is the first recommendation. Caregivers are increasingly becoming a crucial part of the care team. Involving them and providing educational resources to train them for their roles can reinforce their skills and knowledge. It is also essential to monitor their health and the risk of overload and burnout [26]. In the study by Sousa and collaborators (2024) [27], it is described that the low level of education and absence of participation in educational activities by the caregivers may interfere with the caregiver’s contribution to self-care.

One of the recommendations may play a key role in controlling cardiovascular risk factors, which aligns with the predicted increase in the prevalence of cardiovascular diseases over the coming decades. Examples of these risk factors are diabetes, with a rise of 39.3%; hypertension, with an increase of 27.1%; dyslipidemia, with a rise of 27.6%; and obesity, with an increase of 18.3% [28]. By reducing modifiable risk factors and preventing the progression of cardiovascular disease, mortality can be reduced and quality of life improved [29]. Primary, secondary, and tertiary prevention aims to achieve these goals by identifying risk factors to guide lifestyle changes, such as blood pressure control, stress management, and physical activity [29,30]. CR can be a primary caregiver intervention in its educational component, as described in recommendations 2 and 4.

The fifth recommendation reflects the importance of integrating the caregivers to increase the cardiac patients’ adherence to CR programs. The caregivers’ support can facilitate recovery and transition after an acute cardiac event. Integrating the patient-caregiver dyad into CR programs offers scope for developing a better understanding of the influences between partners on recovery over time and more appropriately defining interventions designed to support the patient and caregiver in improving their health [10,31].

Concerning the phases of CR, the sixth recommendation suggests that Phases II and III may be the most fruitful for integrating the caregiver. This is most likely related to the fact that the patient is in a phase of clinical stabilization and that their presence will be more accessible from a care organization perspective. However, it is essential to reiterate that despite the lack of robust evidence regarding the integration of the caregiver in phase I, their presence during hospitalization is a social determinant that affects the patient’s health outcomes, capacity for self-care, and prognosis [32,33].

Telerehabilitation also emerges as a method of integrating caregivers into CR programs, which aligns with what Falter and colleagues described [5]. These authors consider CR a therapeutic modality that can benefit from digital health integration. Since only some institutions implement CR programs, and adherence is low, optimizing these resources is urgently needed.

## 5. Strengths and Limitations of the Study

This study is the first to develop a consensus of CR program components/interventions that can be implemented with caregivers. These recommendations provide an overview of the evidence and represent a tool for professionals to adapt to their clinical practice contexts when integrating the caregiver into cardiac rehabilitation programs.

The existing evidence in this field is of moderate to low quality and indicates the need for more research to identify the components of CR that caregivers can integrate. For this reason, most of the recommendations are based on observational studies, expert opinions, intervention protocols, and reviews. This is the main limitation of this study, which may limit its applicability.

Another limitation is the somewhat subjective process of selecting specialists. While specialists are generally considered individuals with knowledge and experience in the subject matter, there are additional considerations, such as their scientific contributions to the field. Currently, there is no standardized or objective framework for defining who qualifies as a specialist. The qualitative nature of the research may bring the limitation of not allowing extrapolations.

Caregivers were not involved in the development of these recommendations, and this is recognized as another limitation. The recommendations should be reviewed within five years or if relevant evidence is identified before then.

## 6. Conclusions

A consensus was reached on seven recommendations for CR for the caregivers of patients with heart disease. As the incidence of heart disease rises, the healthcare response must evolve accordingly, with CR as a critical pillar in this effort. The caregivers of patients with heart disease tend to share cardiovascular risk factors. The role of the caregiver also induces cardiovascular risk and should, therefore, be the target of preventive intervention or monitoring.

These recommendations provide a comprehensive framework and emphasize the integration of caregivers as a central focus of care and as essential support for their ailing family members. By delineating key components and interventions, these recommendations lay the groundwork for benchmarking and developing a CR strategy that can be implemented and evaluated by healthcare teams to optimize both the health and the caregiving role of these individuals.

## Figures and Tables

**Table 1:** Professional profile of experts invited to the Delphi panel.

Country	Professional Profile	
Australia	Researcher	**
	Researcher and Nurse	
Brazil	Cardiologist	*
	Exercise physiologist	**
	Nurse	**
	Nurse	**
	Nurse	*
	Physiotherapist	
Bulgaria	PRM physician	*
Canada	Physiotherapist	
China	Nurse	
Croatia	Nurse	
	Nurse	
Czechia	Physiotherapist	
Egypt	Physiotherapist	*
France	Cardiologist	**
Germany	Researcher	
Iran	Exercise physiologist	*
	Nurse	
Italy	Cardiologist	
	Cardiologist	**
	Cardiologist	**
	Nurse	
Japan	Physiotherapist	
Mexico	Cardiologist	*
	Cardiologist	*
Portugal	Cardiologist	
	Cardiologist	
	Nutritionist	
	Nutritionist	
	Rehabilitation Nurse	
	Rehabilitation Nurse	
	Rehabilitation Nurse	
	Researcher and Exercise physiologist	**
Romania	Cardiologist	*
UK	Academic researcher	
	Physiotherapist	**
	Rehabilitation Nurse	
USA	Cardiologist	
	Cardiologist	*
	Cardiologist	

* Participation only in the first round. ** Participation only in the two first rounds.

**Table 2:** Results of each round.

**First Round**	**1—Strongly Disagree**	**2—Disagree**	**3—Neither Agree nor Disagree**	**4—I Agree**	**5—Totally Agree**	**Results**
1. Cardiac rehabilitation programs must involve the dyad person with cardiac disease and the caregiver/family	0%	2.33%	6.98%	16.28%	74.41%	Accepted 90.69%
2. Regardless of the person’s cardiac disease, the caregiver benefits from being integrated into the cardiac rehabilitation program.	0%	0%	11.63%	23.26%	65.11%	Accepted 88.37%
3. Caregivers of patients with heart disease benefit from “Health education” sessions, which integrate information about the disease, treatments/medication, diet, and risk factors	0%	2.2%	0%	19.6%	78.3%	Accepted 97.9%
4. Often, patients and caregivers share cardiovascular risk factors, so intervention aimed at both is opportune	4.3%	19%	13%	32.6%	50%	Accepted 82.6%
5. Physical exercise can also be prescribed for the caregiver	2.2%	8.7%	23.9%	34.8%	30.4%	Rejected 65.2%
6. Cardiac rehabilitation can be, for caregivers, also a primary prevention intervention	2.2%	0%	13%	39.1%	45.7%	Accepted 84.8%
7. The participation of caregivers can increase patient adherence to cardiac rehabilitation programs	0%	0%	0%	26.1%	73.9%	Accepted 100%
8. Home-based programs are the most suitable for integrating caregivers	2.2%	8.7%	30.4%	28.3%	30.4%	Rejected 58.7%
9. The use of cardiac telerehabilitation can expand the possibility of integrating caregivers into CR programs	0%	0%	17.4%	32.6%	50%	Accepted 82.6%
**Second Round**	**1—Strongly Disagree**	**2—Disagree**	**3—Neither Agree nor Disagree**	**4—I Agree**	**5—Totally Agree**	**Results/**
1. Caregivers of patients with heart disease may benefit from cardiac rehabilitation programs and should, therefore, be encouraged to participate alongside their relatives in these programs.	0%	0%	14%	34%	52%	Accepted 87%
2. Caregivers of patients with heart disease may benefit from health education sessions comprising information about the disease, nutrition, physical activity, and basic life support training	0%	0%	3%	10%	87%	Accepted 97%
3. It is likely that caregivers of patients with heart disease s and patients share cardiovascular risk factors and, therefore, they may also benefit from participating in the patients’ risk factor management	0%	7%	10%	21%	62%	Accepted 83%
4. Cardiac rehabilitation can be helpful as a primary prevention intervention for caregivers of patients with heart disease.	0%	3%	14%	14%	69%	Accepted 83%
5. The integration of caregivers in cardiac rehabilitation programs is likely to increase the adherence of cardiac patients to programs and, therefore, should be encouraged	0%	0%	0%	24%	76%	Accepted 100%
6. Phases II and III of cardiac rehabilitation may be the most suitable moments to integrate caregivers into these programs	0%	3%	3%	45%	48%	Accepted 93%
7. Telerehabilitation may be a methodology to facilitate the integration of caregivers in cardiac rehabilitation programs	0%	7%	10%	48%	34%	Accepted 82%
** Third Round **	** Comments/Suggestions **					**Class of Recomendadion**
1. Caregivers of patients with heart disease may benefit from cardiac rehabilitation programs and, therefore, should be encouraged to participate alongside the heart disease patient they support in cardiac rehabilitation programs	Suggested change—“therefore should be encouraged to participate alongside their relatives in these programs” to “should be encouraged to participate alongside the heart disease patient they support in cardiac rehabilitation programs”.					II
2. Caregivers of patients with heart disease may benefit from health education sessions comprising information about the disease, nutrition, physical activity, and basic life support training.						I
3. It is likely that caregivers of patients with heart disease and patients share cardiovascular risk factors and, therefore, they may also benefit from participating in the patients’ risk factor management						II
4. Cardiac rehabilitation can be helpful as a primary prevention intervention for caregivers of patients with heart disease.						II
5. The integration of caregivers in cardiac rehabilitation programs is likely to increase the adherence of cardiac patients to programs and, therefore, should be encouraged						I
6. Phases II and III of cardiac rehabilitation may be the most suitable moments to integrate caregivers into these programs						II
7. Telerehabilitation may be a methodology to facilitate the integration of caregivers in cardiac rehabilitation programs	Suggest changing “ methodology” to “method”.					II

**Table 3:** Internacional Delphi consensus results.

Recommendations	Class of Recommendation
1. Caregivers of patients with heart disease may benefit from cardiac rehabilitation programs and should be encouraged to participate alongside the patients they support in such programs.	IIb
2. Caregivers of patients with heart disease may benefit from health education sessions comprising information about the disease, nutrition, physical activity, and basic life support training.	I
3. It is likely that caregivers of patients with heart disease and patients share cardiovascular risk factors and, therefore, they may also benefit from participating in the patients’ risk factor management.	IIb
4. Cardiac rehabilitation can be helpful as a primary prevention intervention for caregivers of patients with heart disease.	IIa
5. The integration of caregivers into cardiac rehabilitation programs is likely to increase the adherence of cardiac patients to programs and, therefore, should be encouraged.	I
6. Phases II and III of cardiac rehabilitation may be the most suitable for integrating caregivers into these programs.	IIb
7. Telerehabilitation may be a method to facilitate the integration of caregivers in cardiac rehabilitation programs.	IIb

## Data Availability

Data is contained within the article The original contributions presented in the study are included in the article, further inquiries can be directed to the corresponding author.
